# Using a quadratic parameter sinusoid model to characterize the structure of EEG sleep spindles

**DOI:** 10.3389/fnhum.2015.00206

**Published:** 2015-05-05

**Authors:** Abdul J. Palliyali, Mohammad N. Ahmed, Beena Ahmed

**Affiliations:** Electrical and Computer Engineering Program, Texas A&M University at QatarDoha, Qatar

**Keywords:** sleep spindles, sleep spindles model, sleep spindle structure, sleep stages, sleep spindle morphology

## Abstract

Sleep spindles are essentially non-stationary signals that display time and frequency-varying characteristics within their envelope, which makes it difficult to accurately identify its instantaneous frequency and amplitude. To allow a better parameterization of the structure of spindle, we propose modeling spindles using a Quadratic Parameter Sinusoid (QPS). The QPS is well suited to model spindle activity as it utilizes a quadratic representation to capture the inherent duration and frequency variations within spindles. The effectiveness of our proposed model and estimation technique was quantitatively evaluated in parameter determination experiments using simulated spindle-like signals and real spindles in the presence of background EEG. We used the QPS parameters to predict the energy and frequency of spindles with a mean accuracy of 92.34 and 97.73% respectively. We also show that the QPS parameters provide a quantification of the amplitude and frequency variations occurring within sleep spindles that can be observed visually and related to their characteristic “waxing and waning” shape. We analyze the variations in the parameters values to present how they can be used to understand the inter- and intra-participant variations in spindle structure. Finally, we present a comparison of the QPS parameters of spindles and non-spindles, which shows a substantial difference in parameter values between the two classes.

## Introduction

Spindles are rhythmic transients present in the electroencephalogram (EEG) characteristic of stage two sleep. Though varying definitions of spindles exist in literature, the American Academy of Sleep Medicine (AASM) has standardized them by describing spindles as “oscillatory bursts on EEG, of 11–16 Hz sinusoidal waves, with a duration of 0.5–2 s and waxing and waning envelope” (Rechtschaffen and Kales, 1968; Iber et al., 2007).

Sleep spindles are used to aid sleep staging (Rechtschaffen and Kales, 1968; Iber et al., 2007). Recent research has shown that they play a role in memory formation and sleep “stability” (Weia et al., 1999; Fogela and Smith, 2011). They have also been found to have an association with various pathological phenomenon such as depression, epilepsy, Parkinson, Alzheimer, and schizophrenia, further raising their significance (Bódizs et al., 2009; Wamsley et al., 2012; Tezer et al., 2014). For example in Fogela and Smith (2011) the authors propose that spindles can be used as possible physiological markers of intellectual ability; spindle properties were found to be highly correlated with tests of intelligence such as IQ tests. The authors also discuss the role of spindles in the consolidation of declarative memory by aiding the interaction between the hippocampus and the thalamus. Similarly, in Bódizs et al. (2005), the authors showed that the grouping and density of fast spindles correlated positively with mental ability measured from standard Raven Progressive Matrices test. Authors in Tezer et al. (2014) reported a significant decrease in the power and density of spindles before epileptic seizures especially in extra temporal lobe epilepsies. Participants with schizophrenia were also found to have drastically reduced density, number and coherence of sleep spindles (Wamsley et al., 2012).

These analyses require accurate labeling of sleep spindles in EEG recordings, which is time-consuming and error-prone when done manually. Automated spindle detection is thus gathering increasing attention from the research community. As spindles are of sinusoidal nature, characterized by progressively increasing, then gradually decreasing amplitude, most spindle detectors utilize features best suited for sinusoidal functions such as Filter banks, Fast Fourier Transforms, Wavelets, and Matching pursuit (Schönwald et al., 2006; Huupponen et al., 2007; Bódizs et al., 2009). The accuracy of these features however decreases when the frequency content of the background EEG overlaps the spindle range causing an increase in the number of false positives. Automatic sleep spindle detection is also hindered due to fluctuations in the frequency patterns and large inter-individual variability (Campbell et al., 1980; Kunz et al., 2000). However, a more significant issue in the development of accurate sleep spindle detectors is the proper training or tuning of these detectors. The broad AASM definition for sleep spindles leaves the manual marking of spindles in EEG data open to some interpretation, leading to low inter-expert agreement for spindle scoring (Kunz et al., 2000). A study by Wendt et al. found an average intra-expert agreement of 72 ± 7% (κ: 0.66 ± 0.07) and an average inter-expert agreement of 61 ± 6% (κ: 0.52 ± 0.07) (Wendt et al., 2015). Thus, the accuracy of sleep spindle detectors when trained and tested using data scored from a single scorer can fall significantly when tested against data scored by other experts. This also makes it difficult to develop validated assessment criteria for automatic sleep spindle detectors to compare the performance of proposed detectors.

A number of mathematical models have been proposed to better characterize the structure of sleep spindles, thus enabling a better understanding of their structure and facilitating further analysis (Olbrich and Achermann, 2005, 2008; Xanthopoulos et al., 2006; Ktonas et al., 2007; Perumalsamy et al., 2009; Nonclercq et al., 2013). In Olbrich and Achermann (2005), the authors fitted autoregressive (AR) models onto EEG data and used it to analyze oscillatory patterns including spindles. The authors further expanded their work in Olbrich and Achermann (2008) to study the temporal organization of spindles. Though, spindles were detected by studying damping constants of the AR model, no physical characteristics of the spindle were modeled. A similar approach was later proposed in Perumalsamy et al. (2009) where oscillations in EEG including spindles were detected using AR models through surrogate data testing. In Nonclercq et al. (2013), the authors modeled the amplitude and frequency of spindles using bivariate normal distributions. The work, motivated by the widely varying values of spindle properties, used tolerance intervals of normal models to detect spindles. However, it was limited to the detection of spindles and did not model intra-spindle variations of these properties.

**Figure 1:**
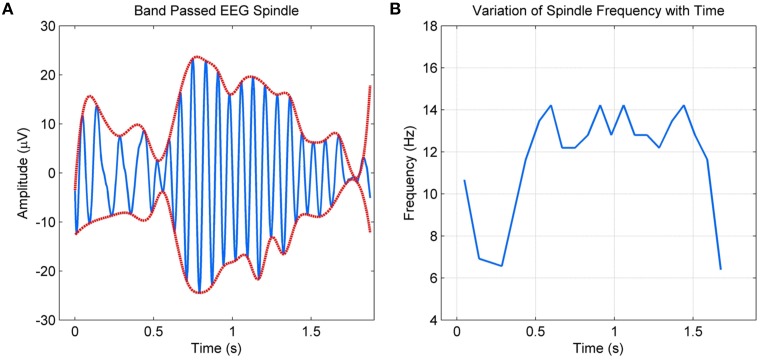
**(A)** Band passed EEG spindle and its envelope **(B)** Non-sinusoidal variation of the spindle frequency with time.

Spindle models as above have been adequate for applications such as the detection of spindles. However, they fail to incorporate details such as the intra-spindle variation of frequencies or “skewness” of the envelope. These details more than often vary with abnormalities or other factors, requiring a model that parameterizes these variations. As spindles have strong amplitude and frequency modulations, non-stationary sinusoidal analysis where the amplitude and frequency are allowed to evolve within the analysis frame are required. In this context, Ktonas et al. (2007) modeled spindles as amplitude and frequency modulated sinusoids. The model consisted of six parameters that captured the time varying microstructure of spindles. The authors also compared various time-frequency analysis methods for parameter estimation in Xanthopoulos et al. (2006) and concluded that complex demodulation provided the best results. They report promising preliminary results with simulated spindles and some selected spindles from three healthy controls and three dementia participants (Ktonas et al., 2009), but do not present detailed validation studies with the model parameters. Furthermore, the sinusoidal form approximation imposed by the model means non-sinusoidal variations in the spindle envelope and instantaneous frequency, as shown in Figure 1, cannot be tracked completely as also discussed by the authors in Ktonas et al. (2009).

In this paper, we extend the work done on spindle modeling using amplitude and frequency modulation with a new Quadratic Parameter Sinusoid (QPS) model to improve the representation of the intra-spindle amplitude and frequency variations without increasing complexity. The model utilizes a quadratic representation to modulate the specific amplitude and frequency variations within spindles. The QPS model was originally used to model non-stationary speech and music (Marques and Almeida, 1989). Non-stationary speech frames were approximated as a sum of time varying frequency and amplitude sinusoids and spectrally analyzed using Short Time Fourier Transforms. The QPS model is well suited to model spindle activity due to its ability to accurately model instantaneous frequency, phase and amplitude in non-stationary signals without the need to assume local stationarity.

The rest of the paper is structured as follows. In the Materials and Methods section we define the QPS model, explain the methodology utilized to estimate the model parameters and experiments conducted to validate the QPS model. We then summarize the results obtained from parameter estimation on simulated spindles with additive white noise and delta EEG as well as real spindles, followed by a discussion of the results and conclusions.

## Materials and methods

### Quadratic parameter sinusoid

Sleep spindles have a waxing and waning sinusoidal form which enables them to be represented as a modulated sinusoidal whose instantaneous frequency and amplitude continuously varies with time. A sleep spindle *s* (*t*) can thus be represented as

(1) $$s\left(t\right)=\;e^{A(t)}cos\;P(t)$$

where *A*(*t*) represents the instantaneous logarithmic amplitude and *P* (*t*) the instantaneous phase. The instantaneous frequency *F*(*t*) can be obtained from the time derivative of *P*(*t*)/2π. Due to the non-stationary nature of EEG, both *A*(*t*) and *F*(*t*) will be time-varying, making their determination non-trivial. For each spindle, as shown in Ito and Yano (1989), both *A*(*t*) and *P*(*t*) can be approximated using Taylor's polynomials around a center time *t_c_*. *P* (*t*) is given by

(2) $$P(t)=\sum_{n=0}^{\infty }p_{n}{\left(t-t_{c}\right)}^{n}/n\text{​}!$$

where,

(3) $$p_{n}=\frac{d^{\left(n\right)}P(t_{c})}{dt^{\left(n\right)}}$$

For frequency to be time-varying, there must be at least one non-zero *p_n_* for *n* ≥ 2 in *P*(*t*). Hence, the minimum possible approximation of *P*(*t*) would be as a quadratic function if the higher order terms are assumed to be negligible. *A*(*t*) can similarly be represented as a quadratic function. This allows the sleep spindle to be defined as a Quadratic-Parameter Sinusoid (QPS) that is given by

(4) $$s\left(t\right)=e^{\left(a\;+\;bt\;+\;ct^{2}\right)}cos\left(d+et+ft^{2}\right)$$

where *a, b, c, d, e* and *f* are the parameters of the quadratic functions *A* (*t*) and *P*(*t*) from (1) respectively. As (4) gives only the real part of the QPS, the general form of *s* (*t*) is given by

(5) $$s\left(t\right)=e^{\left(a\;+\;bt\;+\;ct^{2}\right)}e^{i(d+ft+gt^{2})}$$

**Figure 2:**
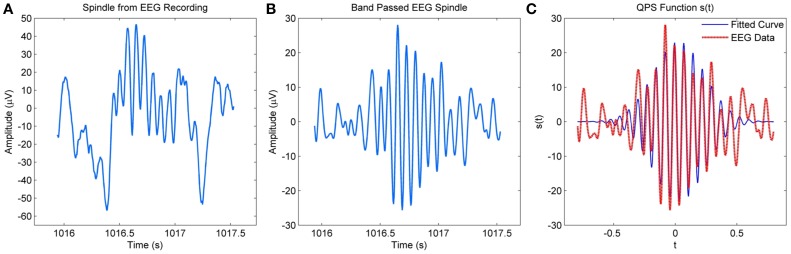
**(A)** Raw spindle from MASS-C1/SS2 EEG recording **(B)** Band-passed version of the EEG spindle **(C)** QPS spindle generated using the parameters of the band–passed version of the spindle.

Figure 2A compares a spindle obtained from an EEG recording to a QPS model generated spindle in Figure 2C. The model was applied to the band passed version of the spindle as shown in Figure 2B. The figure shows considerable similarities between the waxing and waning envelope of the spindle and model.

**Figure 3:**
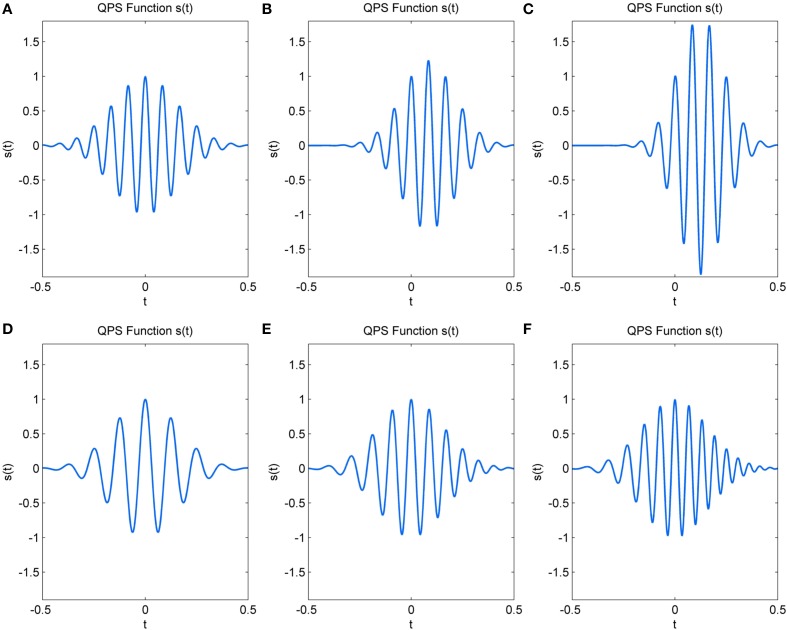
**Change in simulated QPS spindle with (A) *b* = 0, *c* = −20 (B) *b* = 5, *c* = −30 (C) *b* = 10, *c* = −40 (D) *e* = 50, *f* = 0 (E) *e* = 70, *f* = 20 (F) *e* = 90, *f* = 40**.

The 6 parameters (a–f) of the QPS function determine characteristics such as frequency, change in frequency, amplitude, variation in amplitude and the envelope shape of the signal *s*(*t*). The parameters *a, b*, and *c* largely determine the amplitude and the shape of the envelope of the QPS and hence, that of the spindle. *a* is the approximate instantaneous log-amplitude at time, *t* = 0, at which the QPS is centered; *b* the rate of change of amplitude; *c* the Gaussian parameter which determines the shape and duration of the curve (Abe and Smith, 2005). In symmetrical spindles, *b* would be zero, with increasing/decreasing values shifting the time at which the spindle reaches maximum amplitude. Negative values of *c* cause the signal to decay, giving the spindle its rising and waning shape. Figures 3A–C illustrates the variations in amplitude of *s*(*t*) caused by increasing values of *b* and decreasing values of *c*.

The remaining three parameters *d, e*, and *f* influence the frequency characteristics and phase of the signal. *d* represents the initial phase at *t* = 0. The initial frequency of the signal is given by *e*, whereas *f* represents the frequency rate change (Ito and Yano, 2007). In the absence of drastic variations, parameter *e* determines the dominant spindle frequency and *f* causes a linear variation in this frequency within the spindle duration. Figures 3D–F show the variation that occurs in spindle frequency with increasing *e* and *f*.

The highly nonlinear structure of the QPS signal makes parameter estimation of the QPS for a real spindle non-trivial. The problem is further compounded due to the presence of background noise in the EEG. We used non-linear least square (NLLS) estimation using the “Levenberg-Marquardt” technique to obtain the parameters for the QPS model due to its relative simplicity and dependability.

NLLS estimation algorithms are iterative numerical methods that attempt to converge toward optimal parameter values by successively minimizing a sum of squares cost function. The “Levenberg-Marquardt” technique utilized in this work is a standard NLLS implementation that adaptively varies the parameter update between Gradient descent and Gauss-Newton methods using a damping factor. If an iteration results in a large reduction of the cost, the damping factor is decreased bringing the algorithm closer to the Gauss-Newton approach. On the other hand, if an iteration produces negligible cost reduction, the damping factor is increased to mimic a more Gradient-descent strategy. Like all NLLS algorithms, the algorithm can converge to local minima and is heavily dependent on the initial conditions. In our work, convergence was ensured by initializing the parameters to spindle-like values and applying constraints consistent with the AASM spindle definition.

### Experimental validation methodology

Our proposed QPS spindle model was validated using two datasets. The first dataset consisted of a group of simulated spindles with known parameter values. The second dataset consisted of real spindles from the MASS (Montreal Archive of Sleep Studies) database (O'Reilly et al., 2014). This database includes about 1700 h of PSG recording sampled at 256 Hz (O'Reilly et al., 2014). EEG recordings, annotated by two expert scorers, V4 and V5, were retrieved from the 19 participants of MASS-C1/SS2 database. The participants in this subset comprised of 11 women and 8 men with a mean age of 24.3 and 23.2 years respectively and an age range of 18–33 years (O'Reilly et al., 2014). The two expert scorers, V4 and V5 had an average Cohen's Kappa of 0.389 across all participants (O'Reilly and Nielsen, in revision). “It should be noted that relatively low inter-rater agreement is expected between these two scorers since V4 used traditional AASM scoring rules whereas V5 used an approach similar to Ray et al. (2010), O'Reilly and Nielsen (in revision).” Recordings from 4 participants (01-02-0004, 01-02-0008, 01-02-0015, and 01-02-0016) were not scored by V5 as they “were judged reflecting poor quality sleep (e.g., alpha intrusion during N2) or intermittent signal quality/artifact” (O'Reilly and Nielsen, in revision). Hence these recordings were discarded; spindles in the second dataset were thus isolated from the EEG recordings of 15 participants using the annotations of two expert scorers, V4 and V5, with 500 to 1000 spindles per participant. Prior approval for the study was obtained from the TAMU Institutional Review Board.

The accuracy of parameter estimation by the NLLS estimation algorithm was first validated on a simulated spindle dataset, as artificial spindles provided known reference values allowing errors to be quantified. Next, the robustness of the NLLS to varying levels of additive noise was quantified using “Goodness of Fit” measures. White Gaussian noise with wide-ranging SNR values and EEG segments consisting of strong delta components (representative of background EEG) were added to a number of simulated spindles. The QPS parameters of the resultant noisy signal were obtained using NLLS and compared with the parameter values of the original QPS prior to addition of noise.

The performance of the model with real EEG data in MASS-C1/SS2 was then evaluated by determining the error in estimated spindle frequency and energy for spindles marked by both the expert scorers individually and the common spindles marked by both scorers. Trends in the distribution of parameter values across all participants were also analyzed to obtain a better understanding of how spindle structure varied across the participants and how spindles marked by two scorers affect the distribution of these parameter values. The impact of each QPS parameter on the overall spindle shape was also studied by tracking variations in parameter values over the spindle value range. Finally, the ability of QPS parameter values to differentiate between spindles and non-spindle EEG activity was analyzed by comparing parameter values for sample non-spindle and spindle EEG regions in MASS-C1/SS2 database. The results from each of the above validation experiments are detailed in the next section.

## Results

### Validation of QPS model on simulated spindles

#### Accuracy of parameter estimation

**Figure 4:**
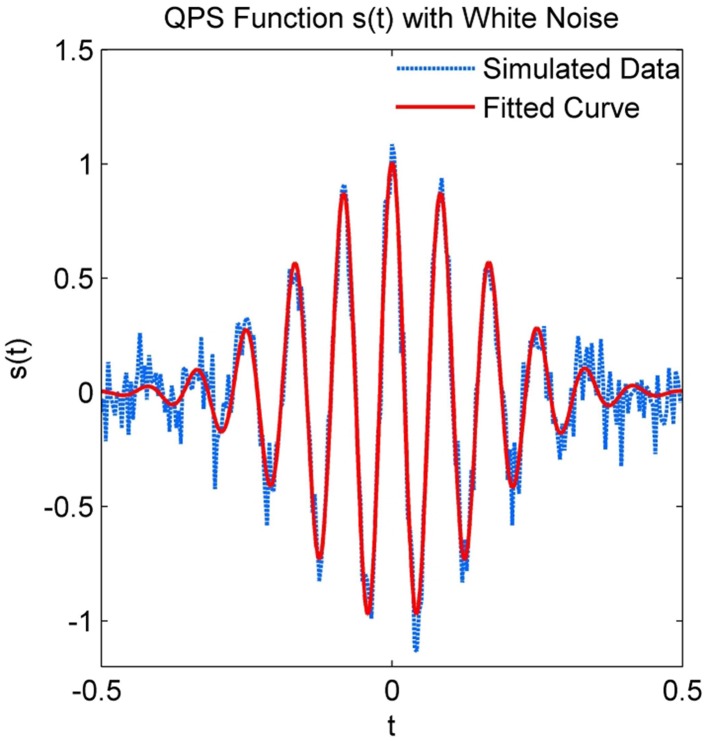
**Simulated QPS spindle with white Gaussian noise and the predicted QPS spindle using estimated parameters**.

The parameters of a simulated spindle with added white Gaussian noise at an SNR of 10 dB were estimated using the NLLS algorithm. Both the true and estimated parameter values are given in Table 1, with the estimated parameters matching the true values within a narrow confidence interval. Figure 4 illustrates the estimated signal (shown in red) superimposed on the noisy signal (shown in blue).

**Table 1:** **True and estimated parameters for a simulated spindle**.

**Parameter**	**True value**	**Estimated value**	**Confidence bounds**
*a*	0	0.003995	(−0.01843, 0.02642)
*b*	0	0.01055	(−0.1505, 0.1716)
*c*	−20	−19.35	(−20.35, −18.35)
*d*	0	−0.01817	(−0.0406, 0.004255)
*e*	75	75.03	(74.87, 75.19)
*f*	0	0.6464	(−0.3566, 1.649)

#### NLLS performance in the presence of white Gaussian noise

We computed the following goodness of fit (GOF) measures on five simulated spindles with spindle like parameter values and varying SNR values:

1. Sum of Squared Errors (SSE)
  (6) $$SSE=\sum_{i=1}^{n}{\left(s_{i}-{\hat{s}}_{i}\right)}^{2}$$
  where, *s_i_* is the *i*^th^ sample of the original signal, ŝ*_i_* the *i*^th^ sample of the estimated signal and *n* is the number of samples, in our case *n* is 256. An ideal fit will result in an SSE = 0.
2. Rsquare
  (7) $$Rsquare=1-\frac{SSE}{SST}$$
  where, $SST=\sum_{i=1}^{n}{\left(s_{i}-\bar{s}\right)}^{2}$ is the total sum of squares about the mean *s*. Rsquare measures the proportion of variance accounted for by the model and should ideally be 1.
3. Degree of Freedom adjusted Rsquare (Adjusted Rsquare)
  (8) $$AdjRsquare=1-\frac{SSE(n-1)}{SST(v)}$$
  where, *v* = *n* − *m*; *v* is the residual degree of freedom and *m* the number of coefficients, In our case, *m* is 6 and *v* equals 250.
4. Root Mean Squared Error (RMSE)
  (9) $$RMSE=\sqrt{MSE}=\sqrt{SSE/v}$$

**Figure 5:**
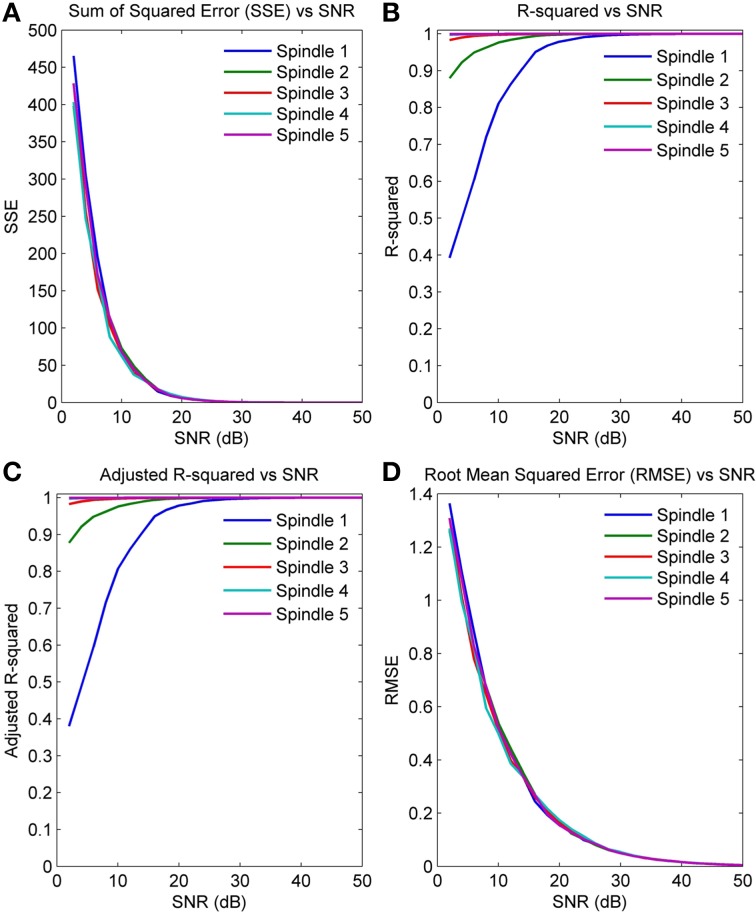
**GOF measures calculated over a range of SNR values for five simulated spindles (A) Sum of Squared Error (B) R-Squared Error (C) Adjusted R-Squared Error (D) Root Mean Squared Error**.

**Figure 6:**
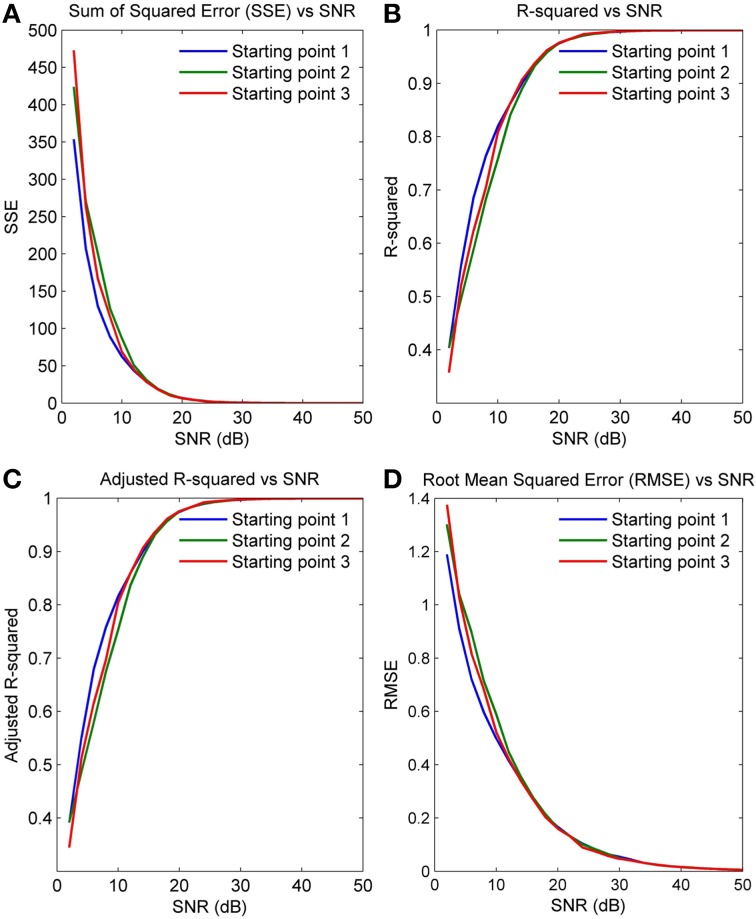
**GOF measures calculated over a range of SNR values for a single spindle but with different initial values for NLLS (A) Sum of Squared Error (B) R-Squared Error (C) Adjusted R-Squared Error (D) Root Mean Squared Error**.

Figures 5A–D plot the four GOF measures for a range of SNRs in the five simulated spindles. As seen, all four GOF measures approach their ideal values with increasing SNR. To determine the impact of the initial parameter values used in the NLLS algorithm on the final converged values, we also executed the NLLS algorithm using a range of different initial conditions for the same spindle. Figures 6A–D show that the parameter estimates still converge at all SNRs despite variation in initial conditions indicating the robustness of NLLS algorithm. As expected, both Figures 5, 6 show that parameters estimated with the NLLS converged to their true values with higher SNR.

#### NLLS performance in the presence of delta noise

**Figure 7:**
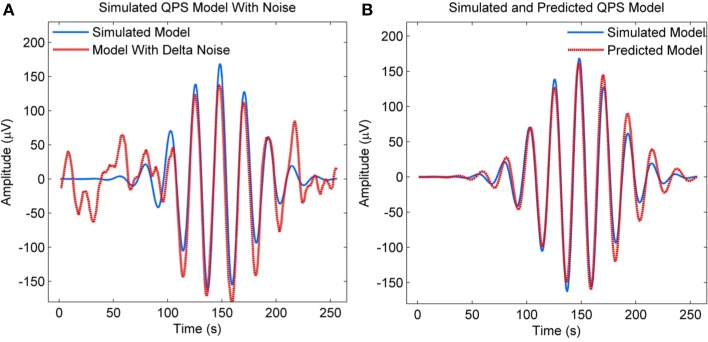
**(A)** Simulated QPS spindle along with the spindle with added delta components **(B)** Simulated QPS spindle along with the predicted QPS spindle from the noisy spindle with added delta components.

We also evaluated the performance of NLLS in estimating QPS model parameters in the presence of strong delta components, since real EEG spindles have these components. The QPS model shown in blue in Figure 7A was simulated using the true parameter values from Table 2. A random EEG segment with delta components was then retrieved from the raw EEG recording of MASS-C1/SS2 participant 1, amplified by a factor of 2 and then added to the simulated QPS model from Figure 7A. The resulting signal is shown in red in Figure 7A. The NLLS algorithm was then used to estimate the parameters of the resulting signal with strong delta components. The QPS model generated using these estimated parameters is shown in red in Figure 7B superimposed on the original simulated noise-free spindle in blue. As seen from Figure 7B, there is no marked difference between the simulated QPS model and predicted QPS model in the presence of delta noise.

**Table 2:** **True and estimated parameter values in the presence of delta noise**.

**Parameter**	**True value**	**Mean estimated value**	**Minimum value**	**Maximum value**
*a*	5	4.990	4.722	5.290
*b*	4	4.122	1.369	8.025
*c*	−30	−29.998	−47.685	−14.420
*d*	1	0.903	−5.363	7.342
*e*	70	70.098	67.138	77.264
*f*	5	4.220	−28.004	20.876

The accuracy of the QPS model parameters in the presence of delta noise was evaluated by adding 190 raw EEG segments with delta components to the simulated model from Figure 7A. The parameter values of the QPS model for these noisy signals were then estimated using NLLS and compared to its actual value. Table 2 shows the mean, minimum and maximum estimated parameter values and the range of estimated values as computed by NLLS in the presence of delta noise. The percentage difference between the true parameter value and the mean estimated parameter value is less than 3% for all parameters except for parameters *d* and *f*, where the percentage difference is 9.7 and 15.6% respectively.

**Figure 8:**
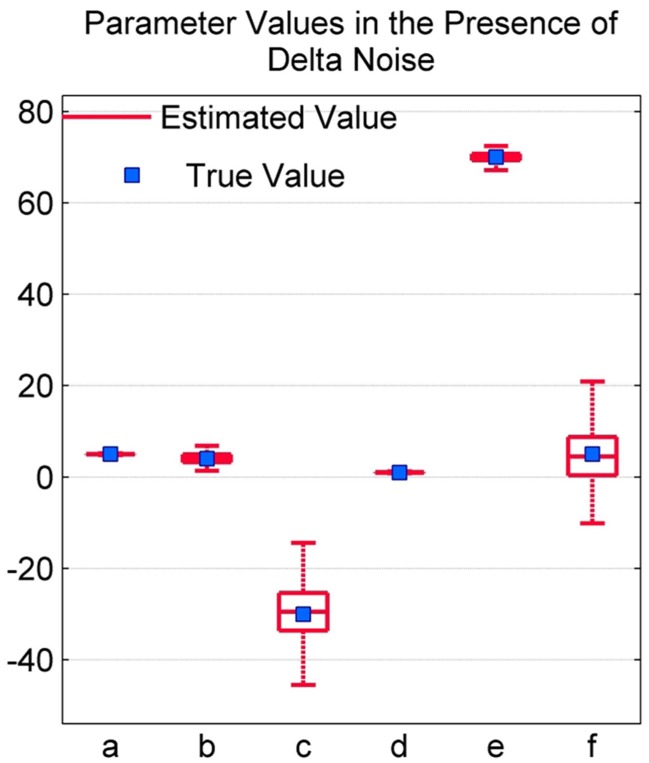
**Boxplot depicting spindle parameter values in the presence of random delta components**.

The boxplot in Figure 8 shows the distribution of estimated parameter values in the presence of delta noise. The true parameter value is indicated using a blue square. The distribution of parameter values is shown using a red box, with the whiskers encompassing ±2.7σ of the data set. As seen from Figure 8 and Table 2, the greatest variation is seen in the values of parameters *c* and *f*, indicating a lower accuracy in estimating these models parameters in the presence of delta noise.

### Validation of QPS model on real spindles

#### Accuracy of energy and frequency estimation

The QPS model was tested on real spindles by estimating model parameters for spindles in the EEG data of 15 participants obtained from the MASS-C1/SS2 database. Since actual spindle parameter values were not known, the QPS model was validated by computing the energy and frequency of the QPS generated spindle and comparing it to the spindle energy and frequency. Energy was calculated by computing the area within the envelope. As the envelope of the QPS generated spindle is given by parameters *a, b*, and *c*, comparing the energy of the generated spindle allowed us to validate the accuracy of these model parameters. The frequency of the QPS generated spindle was obtained from the parameter estimate (*e*/2π) and the spindle frequency obtained from the most dominant peak of the frequency spectrum.

**Figure 9:**
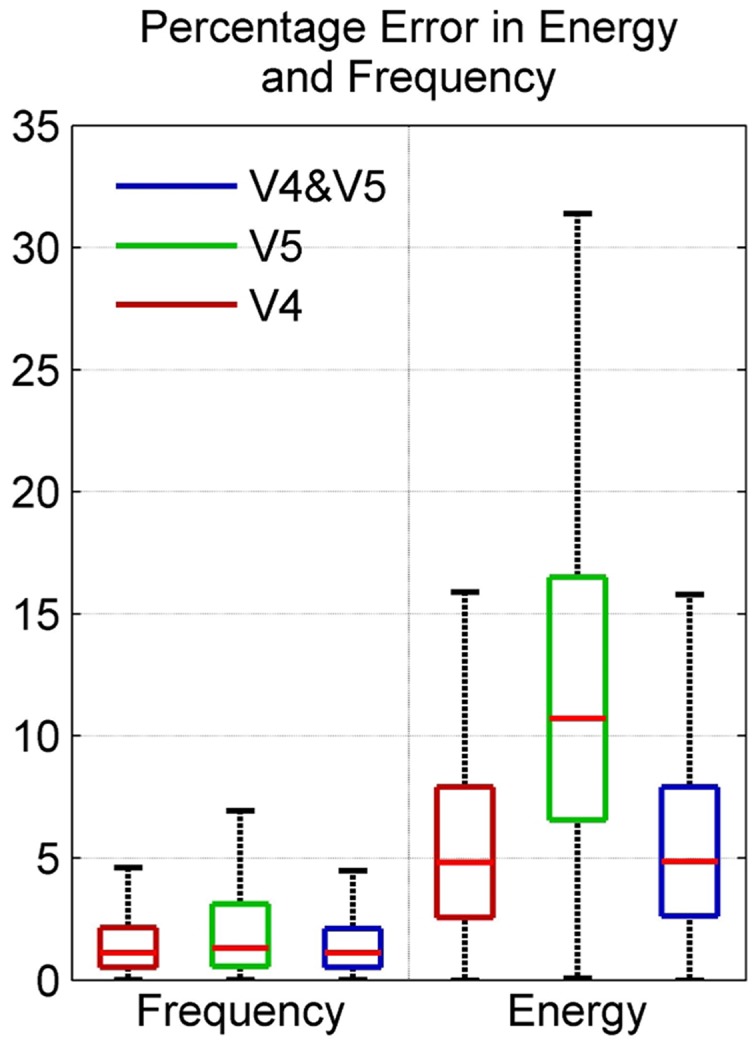
**Box plot depicting percentage error in model energy and frequency using data from MASS-C1/SS2 participants**.

The boxplot in Figure 9 shows the distribution of energy and frequency error using scorers V4, V5, and both V4 and V5; here the whiskers correspond to ±2.7σ. Assuming normal distribution, the frequency error of ~99.3% of the data set is ≤4.6% for scorer V4 and ≤6.9% for V5. The energy error is ≤15.9% for V4 and ≤31.4% for V5. The low variation in frequency error among the scorers as seen in the figure is due to the tighter constraints on frequency values in spindle-marking rule; whereas, the ambiguous definition of spindle amplitude leads to a higher variation in energy error in the two scorers. The subject-specific scoring criteria used by V5 which was based upon each subject's mean peak spindle amplitude meant that their marked spindles fell in a different and narrower amplitude range to the range of spindles marked by V4; this resulted in a low average inter-scorer agreement between V4 and V5 and higher error rate for the QPS model for V5 marked spindles. The relatively low percentage frequency error of the QPS model suggests that it accurately captures the frequency content of spindle. On the contrary, there is a relatively higher energy error as our model attenuates faster than what occurs in actual spindles as seen in Figure 2.

Table 3 shows the mean percentage error in energy and frequency of spindles as scored by scorers V4 and V5 and the mean percentage error in energy and frequency of spindles marked in common by both these scorers. As seen, the overall average mean error in energy and frequency for all participants is the lowest for spindles marked by both the scorers (last row of Table 3). Furthermore, the same observation holds true for the mean error in energy and frequency for most of the individual participants. Reliable spindle scoring is typically achieved by using only spindles marked by multiple scorers. The lower error rate for commonly marked spindles indicate that the QPS model provides an accurate representation of “reliably” marked spindles.

**Table 3:** **Mean percentage error in energy and frequency of spindles**.

**MASS-C1/SS2 Participant #**	**Mean energy error**			**Mean frequency error**		
	**V4**	**V5**	**V4 and V5**	**V4**	**V5**	**V4 and V5**
1	4.204	10.474	4.045	1.379	2.764	1.389
2	5.383	10.606	5.079	1.631	2.198	1.610
3	4.059	4.059	4.114	1.602	1.602	1.730
5	5.150	11.524	5.032	1.866	3.090	1.916
6	4.849	11.866	4.685	1.880	4.668	2.085
7	8.567	15.262	7.907	3.023	3.292	2.197
9	5.510	14.340	5.492	2.249	3.618	1.543
10	6.127	15.683	6.242	2.080	5.092	2.022
11	4.509	11.640	4.798	1.569	2.258	1.522
12	8.209	13.598	7.689	2.138	4.087	2.069
13	5.030	12.380	4.850	2.000	1.902	1.299
14	5.380	13.420	5.469	1.639	2.367	1.561
17	3.568	12.662	3.579	1.640	2.886	2.022
18	8.520	11.478	7.020	2.813	3.356	2.549
19	5.144	10.021	5.362	1.677	2.423	1.795
Average	5.614	11.934	5.424	1.946	3.040	1.821

#### Detailed validation on MASS-C1/SS2 database

**Figure 10:**
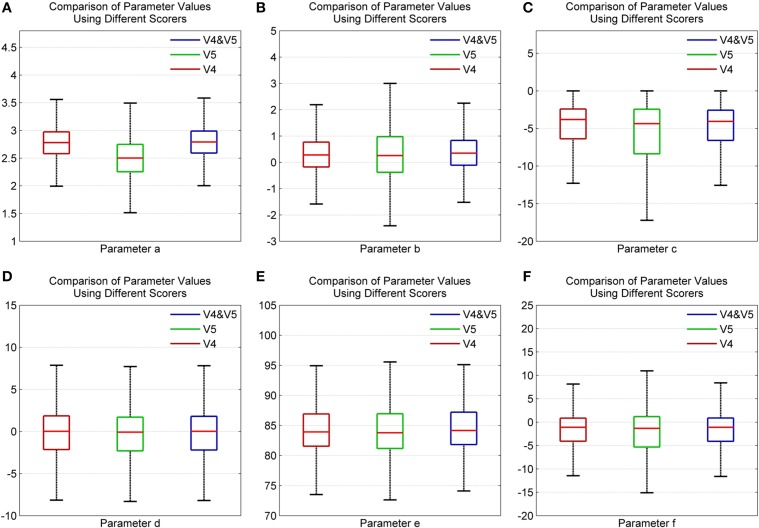
**Box plot depicting the comparison of spindle parameter values using different scorers for parameters **(A)***a***(B)***b***(C)***c***(D)***d***(E)***e* and **(F)***f* using data from MASS-C1/SS2 participants**.

Figures 10A–F show the distribution of parameter values for all participants using scorers V4, V5, and both V4 and V5. Here, the whiskers correspond ±2.7σ of the data set. As seen, parameters *d* and *e* have an identical distribution for both scorers, V4 and V5. This indicates that there is greater agreement among the scorers in the frequency and phase content of the signal. Furthermore, for all parameters, the spindles marked in common by both V4 and V5 show a distribution pattern similar to that of V4. The lower agreement among the scorers regarding spindle amplitude is due to the different scoring criteria used by the scorers. V4 used standard AASM scoring rules while V5 used a subject-specific amplitude threshold to score spindles (Ray et al., 2010).

**Figure 11:**
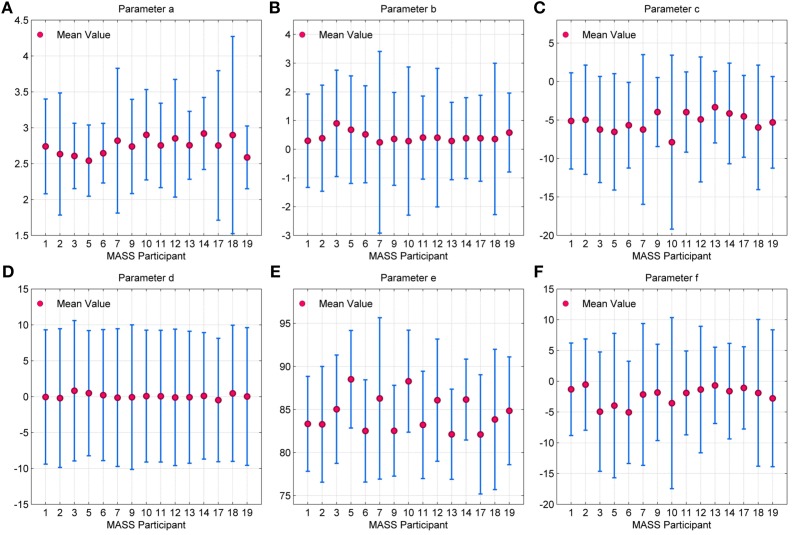
**Error bar depicting the distribution of values for parameters (A) *a***(B)***b***(C)***c***(D)***d***(E)***e* and (F) *f* for each MASS-C1/SS2 participant**. Here, the whiskers represent 2σ.

The error bar in Figures 11A–F, with whiskers representing ±2σ show the distribution of parameter values across all the 15 participants using spindles that have been marked in common by scorers V4 and V5. As seen, the mean values of parameters *a* and *b* fall within the narrow range of (2.5, 3) and (0, 1). Additionally, the mean values for parameter *e* fall within the spindle characteristic frequency range of 11–16 Hz for all participants. The low variance of parameters *a* and *b* for scorers, as given in Table 4, is in line with spindle amplitude and shape scoring criteria. The table also indicates that parameters *c, d, e*, and *f* have the most variation. The variance in *e* is representative of the 11–16 Hz spindle frequency range. Parameters *a, b*, and *e* give spindles the characteristic waxing and waning shape as defined in the AASM guidelines whereas *c, d*, and *f* are more likely to account for the intra- and inter-participant variability in the spindle structure. *d* is more likely to account for the intra-participant variability whereas *c* and *f* could impact the inter-participant variability in the spindle structure.

**Table 4:** **Variance of parameter values for all participants**.

**Parameters**	**V4**	**V5**	**V4 and V5**
*a*	0.195	0.575	0.168
*b*	1.115	3.946	1.087
*c*	14.252	44.246	15.265
*d*	21.727	20.761	22.027
*e*	16.790	20.640	14.928
*f*	25.677	57.356	24.456

### Effect of QPS model parameters on spindle shape

To evaluate the effect of variation of each QPS parameter on the shape of a marked spindle from the MASS-C1/SS2 database, we linearly increased the value of each of the six parameters of the fitted QPS spindle while keeping the value of the other five parameters constant. We thus regenerated new QPS spindles with five constant parameters and a linearly increasing sixth parameter. Instead of choosing an arbitrary constant parameter value, the mean value of the other five parameters of spindles from participant 1 of MASS-C1/SS2 (01-02-0001) scored by scorer V4 was used.

#### Parameter *a*

**Figure 12:**
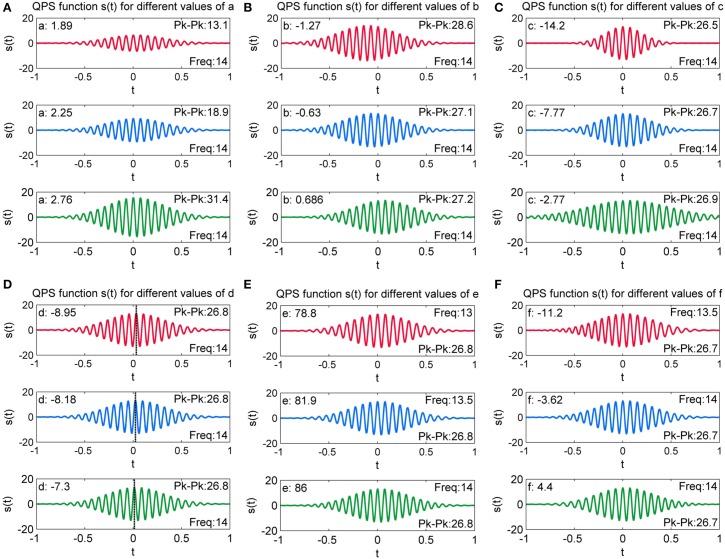
**Variation of QPS spindles with varying values of (A) *a***(B)***b***(c)***c***(D)***d***(E)***e* and (F) *f***. Here, the parameter values are changed incrementally and the resulting effect on the QPS model is observed.

Figure 12A presents the generated QPS spindle for different values of parameter *a*. The plots demonstrate that increasing the value of *a* increases the peak to peak of the generated spindle but does not impact the sinusoidal content of the signal. As *a* increases from *a* = 1.89 to 2.76, the peak to peak value increases from 13.1 to 31.4, thus signifying that the amplitude of generated QPS spindle has a strong positive correlation to the value of *a*.

#### Parameter *b*

Figure 12B shows generated QPS spindles with varying values of parameter *b*. It can be observed that parameter *b* values of −1.27, −0.63, and 0.686 changes the peak to peak value of the generated spindles to 28.6, 27.1 and 27.2 respectively, but the relative change in peak to peak value is not as pronounced as the variation caused by change in *a*. Figure 12B further illustrates that *b* produces asymmetry in the spindle, with the spindle shifting along the time axis.

#### Parameter *c*

Figure 12C presents the generated QPS spindles for different values of parameter *c*. The plots indicate that parameter *c* controls the rate of decay while producing minute variations in the peak to peak value of generated QPS spindles. The decay rate decreases with increasing value of *c*. For instance, Figure 12C shows that the fastest decay rate occurs with the lowest value of *c* (*c* = −14.2), but as *c* approaches 0 (*c* = −2.77), the generated spindle loses its characteristic “spindle-like” shape. Only a small number of spindles in the MASS-C1/SS2 database had values of c approaching 0 (0.5% of all spindles), indicating a low proportion of cases showing a large fitting error.

#### Parameter *d*

Generated QPS spindles with varying values of parameter *d* are shown in Figure 12D. A dashed black line has been added in the individual plots of Figure 12D to indicate the value of *t* at which the spindle attains the maximum peak amplitude value. These plots demonstrate that the variation in parameter *d* induces a phase shift in the generated spindle. Since parameters *a, b, c, e*, and *f* are fixed, all three spindles shown here have the same amplitude and frequency with only the position of the maximal value shifting due to *d* (phase shift).

#### Parameter *e*

The value of parameter *e* and the corresponding QPS model generated spindle can be seen in Figure 12E. The figures indicate that increasing the value of parameter *e* increases the frequency of the generated spindle without affecting its amplitude, thus corroborating that parameter *e* corresponds to the angular frequency of the spindle [see the Accuracy of Energy and Frequency Estimation section]. Discarding outliers, we found all values of e to fall within the characteristic spindle frequency range of 11–16 Hz.

#### Parameter *f*

Figure 12F shows the value of parameter *f* and the corresponding QPS spindle. The initial frequency was fixed at a constant value of 13.2 Hz. As seen here, parameter *f* values of −11.2, −3.62, and 4.4 changes the model frequency to 13.5, 14, and 14 Hz respectively. The figures indicate that increasing the value of parameter *f* induces minor variations in the frequency of generated spindles, thus signifying that the intra-spindle variation in the frequency of the QPS spindle is correlated to the change in *f*.

### Variation of parameter values in an overnight recording

**Figure 13:**
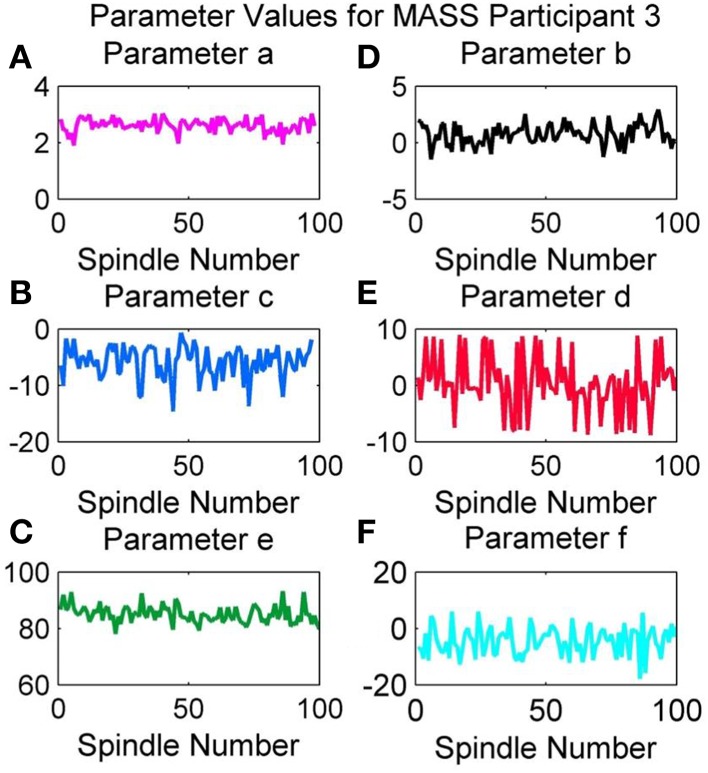
**Variation in values of parameters (A) a, (B) b, (C) c, (D) d, (E) e, and (F) f of MASS-C1/SS2 participant 3 during an overnight recording**.

Figure 13 provides the variation in the QPS parameter values for the spindles marked by scorer V4 in the overnight recording of MASS-C1/SS2 participant 3 (01-02-0003). As expected from the results in Table 4, the least variation over the night's spindles can be seen in parameters *a* and *b*, whereas the most variation is in *c, d, e*, and *f*. Interestingly all parameters show a cyclic rise and fall over the course of the night. Figure 13B shows a decrease in the variation of parameter *b* and increase in its minima during the middle of the recording. Figure 13D also shows a decrease in the variation of parameter *d*, however this occurs later in the recording and is accompanied by a visible dip in the maxima values instead. Parameters *a* and *b* on the other hand show an increase in the peak-to- peak values during the middle of the recording. Figure 13 gives an example of how the QPS parameter values of spindles in an overnight recording can be tracked to better understand the natural physiological variations that can occur during the night.

### Comparison of QPS spindle and non-spindle parameters

In our final experiment, the NLLS algorithm was applied to random non-spindle EEG regions. These were obtained by randomly selecting 500 segments of unmarked EEG data that were 1 second in duration using the two scorers, V4 and V5. The data included all the 15 MASS-C1/SS2 participants and were classified into two groups. The first group contained non-spindles from only sleep stage two (Group 1), whereas the second group contained non-spindles from all sleep stages (Group 2). Special focus was paid to stage two data (Group 1) as spindles are typically observed in EEG during sleep stage two. The resulting set of parameter values given by NLLS were then compared to those obtained from QPS spindles.

Table 5 shows the results from a two-sided non-parametric *t*-test comparing parameter values from QPS spindles and non-spindles using the mean spindle parameter values as obtained in the Detailed Validation on MASS-C1/SS2 Database section. Here, *h* = 0 indicates that the null hypothesis (parameter values from spindles and non-spindles come from distributions with equal means) cannot be rejected at a significance level of 1%. The *p*-value for each parameter is also shown in Table 5. As seen, parameters *a* and *c* were significant at the 0.01 level for both scorers and the two groups. With Group 2 non-spindles, *b, e*, and *f* were also significantly different from spindles for scorer V5 but not for V4.

**Table 5:** **Results of two sided *t*-test comparing parameters obtained from QPS spindles and non-spindles using same initial conditions**.

**Parameters**	**V4 (Group 2)**		**V5 (Group 2)**		**V4 (Group 1)**		**V5 (Group 1)**	
	***h***	***p***	***h***	***p***	***h***	***p***	***h***	***p***
*a*	1	0	1	0	1	0	1	0
*b*	0	0.112	0	0.052	0	0.263	1	0
*c*	1	0	1	0	1	0	1	0
*d*	0	0.051	0	0.429	0	0.559	0	0.379
*e*	0	0.809	1	0.003	0	0.550	1	0
*f*	0	0.063	1	0	0	0.011	1	0

Table 6 shows the results from a two-sided non-parametric *t*-test using different sets of initial conditions for spindles and non-spindles. Given the wide range of possible non-spindle waveforms, the NLLS was initiated with all parameters = 0 for non-spindles, whereas the NLLS was initialized with the mean spindle parameter values for spindles. As seen in Table 6, all parameters show significant difference for both the scorers and the two groups; with the only exception being Group 1 non-spindles for parameter *c* of scorer V4.

**Table 6:** **Two sample *t*-test result comparing parameters obtained from QPS spindles and non-spindles using different initial conditions**.

**Parameters**	**V4 (Group 2)**		**V5 (Group 2)**		**V4 (Group 1)**		**V5 (Group 1)**	
	***h***	***p***	***h***	***p***	***h***	***p***	***h***	***p***
*a*	1	0	1	0	1	0	1	0
*b*	1	0	1	0	1	0	1	0
*c*	1	0.003	1	0	0	0.331	1	0
*d*	1	0	1	0	1	0	1	0
*e*	1	0	1	0	1	0	1	0
*f*	1	0	1	0	1	0	1	0

The dependency of the NLLS on the initial conditions limits the parameters of QPS function from accurately differentiating between spindles and non-spindles, as seen from the results in Tables 5, 6. We expected a significant difference in parameter *e* values for spindles and non-spindles. However when the initial value of parameter *e* for both non-spindles and spindles was set at 90, the value corresponding to the spindle frequency, parameter *e* values for non-spindles converged to a local minimum close to that value; significant differences in parameter *e* values could thus not be observed in the results from scorer V4. The difference in parameters *a* and *c* for all the groups using both scorers indicates significant difference in the amplitude variations of QPS spindles and non-spindles. Using different initial conditions for non-spindles resulted in significantly differences for all parameter values from those of spindles.

## Discussion and conclusions

In this paper we proposed a new method to model the instantaneous frequency and amplitude variations occurring within sleep spindles. Our proposed QPS model is able to account for the non-stationarity observed in sleep spindles within the analysis window by accurately approximating the frequency and logarithmic amplitude of the signal using quadratic functions of time. Our results illustrate that QPS successfully models the various intra-spindle characteristics within its six parameters. Parameter estimation using standard NLLS methods resulted in good convergence and was robust in the presence noise, both of which are vital given the presence of background EEG. The relative error in frequency estimates was less than 5% when compared to the dominant peak in the spindle frequency spectrum for a majority of the participants.

The reversibility between the determined parameters and signal waveform is also an important characteristic of the QPS modeling. As seen in Figures 4, 7, it is possible to regenerate a cleaner version using the QPS parameters. Unlike other techniques, the QPS model also provides the instantaneous phase, which is indispensable in signal reconstruction. The results in the Validation of QPS Model on Simulated Spindles section show that it possible to use the QPS to regenerate cleaner versions of spindles in EEG with large artifacts and background noise. The noise component identified in the spindles could then be used to de-noise adjacent areas of the sleep EEG.

Characterizing sleep spindles using the QPS parameters could help restrict the inconsistency in scoring due to the differing subjective interpretation of scorers, which will in turn assist in the proper training and tuning of accurate sleep spindle detectors. As seen in Table 4, parameters *c, d*, and *f* had the most variation. The broad AASM definition for sleep spindles currently leaves the manual marking of spindles in EEG data open to some interpretation, leading to low inter-expert agreement for spindle scoring. Thus, the accuracy of sleep spindle detectors when trained and tested using data scored from a single scorer can fall significantly when tested using data scored by other experts. Spindle scoring reliability is typically reduced by having multiple scorers detect spindles manually and accepting only commonly marked spindles. The frequency (1.8%) and energy (5.4%) error estimates for the QPS model were lowest for the spindles marked by both scorers (Table 3), indicating that it provides a more accurate representation of the “reliably” marked spindles. Providing guidance on an acceptable range for all QPS parameters in the spindle scoring criteria using spindles marked by multiple scorers can help reduce scoring inconsistencies.

Accurately characterizing the structure of sleep spindles could enable researchers develop a better understanding of the relationship between sleep spindles and various physiological phenomena such as sleep “stability,” memory formation and other pathological problems, e.g., depression, epilepsy, Parkinson, Alzheimer and schizophrenia (Weia et al., 1999; Bódizs et al., 2005; Fogela and Smith, 2011; Wamsley et al., 2012; Tezer et al., 2014). The relationship of spindle amplitude and frequency, from parameter a and *e* with these phenomena have been researched. However, their impact on the rate of decay of the spindle envelope (*c*), the phase shift (*d*) and frequency variation (*f*) have not been studied to date. The QPS parameters offer quantitative representations of spindle structure that can be interpreted visually, as presented in the Effect of QPS Model Parameters on Spindle Shape section. Variations in these parameters can be analyzed to determine if they are disorder, scorer or participant specific.

Additional potential uses of the QPS model include the generation of a wide range of simulated spindles to help accurately train automatic detectors as well as manual scorers. The simulated QPS spindles can also be utilized to provide a reference to define more precise scoring rules, normalize real spindles from multiple participants and also compare real spindles against to track naturally or pathologically occurring variations.

The similarity in distribution patterns and limited range of the QPS parameter values (Figure 11) indicate that there is potential in their use in an automatic spindle scoring algorithm. The results in the Comparison of QPS Spindle and Non-Spindle Parameters section however show that NLLS estimation are highly dependent on the initial conditions used. The parameter values showed significant difference between the two groups when different initial conditions were used for spindles and non-spindles. However when the same initial conditions were used for both groups, surprisingly only the amplitude based parameters *a* and *c* were significant and not the frequency based *e*. These results indicated that the QPS model can only be used for spindle detection if preceded by a priori parameter estimation to obtain the initial conditions to be used in the NLLS for each epoch or an alternative QPS parameter estimation technique, e.g., an analytical method, that does not depend on initial conditions is utilized instead of the NLLS algorithm.

In this study, parameter estimation was performed using the NLLS algorithm. Results obtained with NLLS need to be compared with other parameter estimation techniques. Furthermore, as discussed above NLLS results can depend on the chosen initial conditions. Like other recursive methods, the NLLS algorithm can be computationally expensive. Future work will include identifying more robust algorithms for parameter estimation including analytical methods, thus overcoming the burden of initial conditions and ensuring global convergence. Simplified as well as expanded versions of the QPS model with more parameters also need to be explored as they may enhance the characterization of spindle structure.

We also intend to use the QPS parameters to develop an accurate sleep spindle detection algorithm, taking into account the limitations stated above and test it on spindles from the MASS-C1/SS2 database. The accuracy of the automated detector will be compared to existing spindle detector available through the Spyndle toolbox. Finally, as mentioned earlier, the QPS model opens up the potential to examine in detail the impact of sleep abnormalities and disorders as well as other physiological processes on sleep spindles.

### Conflict of interest statement

The authors declare that the research was conducted in the absence of any commercial or financial relationships that could be construed as a potential conflict of interest.
